# The Low Breaking Fiber Mechanism and Its Effect on the Behavior of the Melt Flow of Injection Molded Ultra-Long Glass Fiber Reinforced Polypropylene Composites

**DOI:** 10.3390/polym13152492

**Published:** 2021-07-28

**Authors:** Po-Wei Huang, Hsin-Shu Peng, Sheng-Jye Hwang, Chao-Tsai Huang

**Affiliations:** 1 Program of Mechanical and Aeronautical Engineering, Feng Chia University College of Engineering and Science, Taichung 40724, Taiwan; bowei8915@gmail.com; 2 Department of Mechanical and Computer Aided Engineering, Feng Chia University College of Engineering and Science, Taichung 40724, Taiwan; 3 Department of Mechanical Engineering, National Cheng Kung University College of Engineering, Tainan 70101, Taiwan; jimppl@mail.ncku.edu.tw; 4 Department of Chemical and Materials Engineering, Tamkang University College of Engineering, New Taipei City 251301, Taiwan; cthuang@mail.tku.edu.tw

**Keywords:** fiber breaking behavior, flow ability, plasticizing properties, polypropylene, ultra-long-fiber composite

## Abstract

In this study, fiber breaking behavior, fiber orientation, length variation, and changes in melt flow ability of long glass fiber reinforced polypropylene (L-FRP) composites under different mold cavity geometry, melt fill path, and plasticization parameters were investigated. The matrix material used was polypropylene and the reinforcement fibers were 25 mm long. An ultra-long-fiber composite injection molding machine (with a three-stage plunger and injection mechanism design) was used with different mold cavity geometry and plasticization parameters. Different screw speeds were used to explore the changes in fiber length and to provide a reference for setting fiber length and parameter combinations. Flow-length specimen molds with different specimen thickness, melt fill path, and gate design were used to observe the effect of plasticizing properties on the flow ability of the L-FRP composite materials. The experimental results showed that the use of an injection molding machine with a mechanism that reduced the amount of fiber breakage was advantageous. It was also found that an increase in screw speed increased fiber breakage, and 25 mm long fibers were shortened by an average of 50% (to 10 mm). Long fibers were more resistant to melt filling than short fibers. In addition, the thickness of the specimen and the gate design were also found to affect the filling process. The rounded angle gate and thick wall product decreased the flow resistance and assisted the flow ability and fiber distribution of the L-FRP injection molding.

## 1. Introduction

Long-fiber reinforced polymer (L-FRP) composite injection molding has gained significant attention in recent years because of its many favorable characteristics, such as lightweight, low cost, good stiffness, high tensile modulus, and superior impact strength [1,2]. Broadly speaking, the mechanical behavior of L-FRPs depends on three main factors: average fiber length, fiber orientation, and fiber concentration [3,4]. Over the last decade, L-FRPs have been intensely studied and are now widely used in the industry [5,6,7]. There are two commonly used types of processing technology used for glass fiber reinforced polymer composites: short glass fiber and continuous L-FRP composites [8,9]. L-FRP composites provide a better mechanical performance than short-fiber reinforced polymers (S-FRP). However, the fibers can easily be damaged when the L-FRPs material is moved through a plasticization screw [10], and the injection molding of L-FRPs is not an easy job. This is made more complicated by the fact that the fiber orientation is also determined by the flow process during injection. The fibers can also suffer damage when the polymer melt moves through mold cavities because of poor gate, runner, or cavity design. Inappropriate parameter settings may also cause the formation of defects such as fiber bundles and air traps, which can further degrade the mechanical properties of the product. The preservation of the fiber integrity and dense uniform orientation of the fibers within the final product are critical to high quality injection molding. Injection-molded L-FRP specimens typically have a skin/shell/core structure [11], and fiber orientation in the loading direction is important for the modulus and strength of L-FRP composites. The degree of fiber alignment in the flow direction is also sensitive to the fiber weight fraction [12,13]. Many studies have been done on the effects of the parameters on the properties of L-FRP specimens, and it was found that a higher-melt temperature is beneficial in reducing the viscosity of the polymer melt and improving the uniformity of the fiber dispersion within the matrix. However, a high screw speed and a low feed rate increase the shear rate acting on the polymer melt and lead to more serious fiber breakage while improving the mixing properties [14,15]. Rohde et al. showed that the back pressure has a detrimental effect on both the fiber length and the impact energy absorbance of L-FRP specimens [16]. Kumar et al. investigated the effects of the injection pressure and screw speed on two thermoplastics compounded with different initial fiber lengths [17,18,19,20]. The results showed that an understanding of the orientation behavior of the fibers in the injection molding process is critical. In the flow process during injection molding, the final layer structure is determined by the fiber orientation, length after breaking, and the proportion of the fiber flowing into the product. The results of this research can be applied to the manufacture of automobile and aircraft components that need both mechanical strength and a high tensile modulus.

In this study, Equation (1) was used to examine the fluidity and the flow behavior of the melt, the effect of the volume of the mold cavity on the melt, and the through flow-length of the specimen. It allowed for the calculation of the melt flow length ratio variations and the flow ability of injection molding. The melt flow length ratio (L/t) can be used not only as a guide for determining the gate location and balancing the melt flow within the mold cavity, but also as a means of measuring the melt fluidity and testing the performance of the injection machine [21,22]. Equation (1) was also used to investigate the plasticizing and molding parameters involved in the injection molding of long glass fiber composites. The melt flow length ratios to part thickness also needed to be determined. In the equation, “L” is the total melt flow length and “t” is the average thickness. L_1_/t_1_, L_2_/t_2_, L_3_/t_3_, and L_4_/t_4_ are the melt flow length ratios of the cold slug well, runner, gate, and cavity, respectively. The melt flow length ratio (L/t) can be used as a guide for the determination of the gate location and to balance the melt filling within the cavity. A higher flow length ratio or greater filling distance yields a more favorable melt filling property.

(1) $$\frac{L}{t}=\frac{L_{1}}{t_{1}}+\frac{L_{2}}{t_{2}}+\frac{L_{3}}{t_{3}}+\frac{L_{4}}{t_{4}}$$

The high mechanical performance characteristics of the long glass fiber reinforced polypropylene (L-FRP) composites are the reason they are often selected as substitutes for metals, as a replacement for underperforming plastics, or as alternatives to higher-cost engineering polymers through the up-engineering of lower-cost plastics. There is a clear relationship between tensile stress, the plasticizing process, and changes of fiber length in L-FRP. In this study, a special ultra-long fiber composite injection molding machine was developed and used with different screw speeds in order to investigate the fiber breaking. The molding material used was an L-FRP composite with 25 mm length fibers. The specimens used were 1 mm and 3 mm thick and the spiral flow mold cavity, with a new melt fill path and gate design, was made specially to observe the changes in the fiber breaking and flow ability. The low fracture fiber mechanism and specially designed injection molding system could retain the fiber length to a significant degree. An experiment was also conducted to study the melt flow length of the fiber by varying several plasticizing parameters, namely screw speed, back pressure, melt temperature, and mold temperature, in order to evaluate the changes in the melt flow length of the L-FRP specimens.

## 2. Experiment Methods

### 2.1. Design of Specimens and Mold

**Figure 1:**
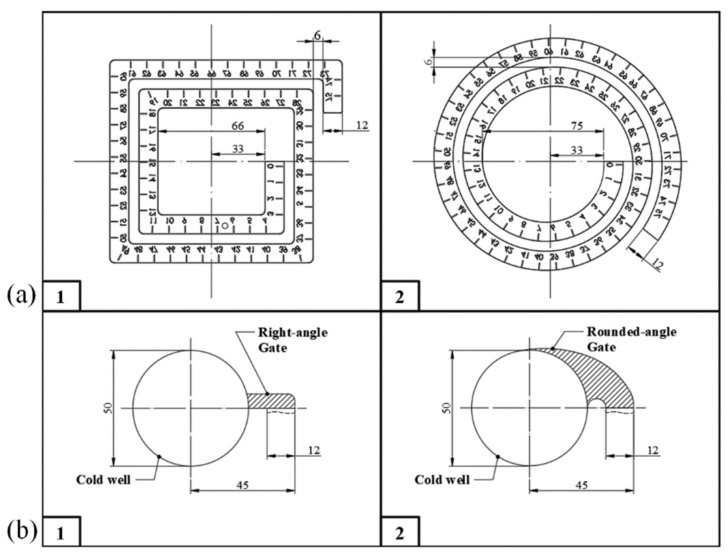
(**a**) Schematic of the flow length specimens (1 mm and 3 mm thick) and different melt fill paths: (**1**) square and (**2**) round. (**b**) Flow length specimens with different gate designs: (**1**) right-angular and (**2**) rounded-angle.

To observe the flow ability of the long fiber reinforced polypropylene composites, special injection test specimens and molds were designed, the mold was developed jointly with Der-Li industry Works Co. Ltd. (Tainan City, Anding District, Taiwan) To investigate the differences in the fiber breaking and melt flow ability, a spiral flow mold design was used (see Figure 1a). Different melt fill paths (square and round) were used to measure the fiber/melt fluidity and melt flow length ratios. In the experiment, the volume was fixed by a screw position of 20 mm. The melt flow length was measured directly, and the melt flow length ratio to the part thickness was calculated using Equation (1). The specimens were 1 mm or 3 mm thick and the gate was either right-angular or rounded-angle, as shown in Figure 1b.

### 2.2. Material and Molding Equipment

**Figure 2:**
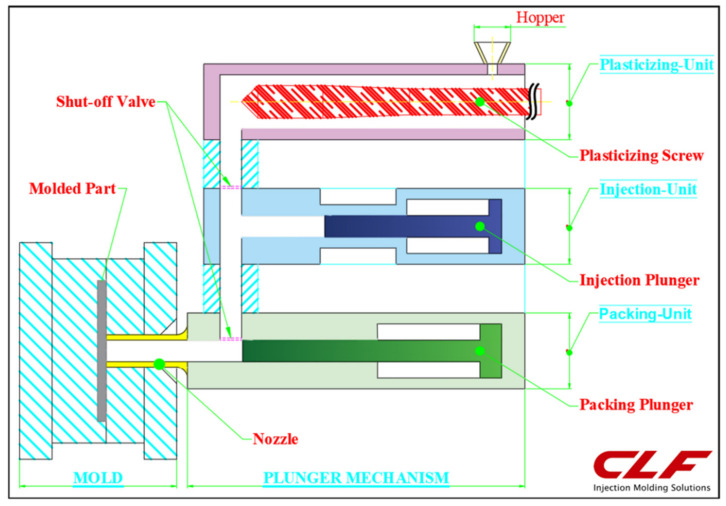
Diagram of the jointly developed ultra-long fiber injection machine with a special three stage plunger and low breaking fiber mechanism.

The composite material used in this study was LGP50-3 (50 wt.% glass fiber in polypropylene) manufactured by the Great Eastern Resins Industrial Co. Ltd. (Taichung City, Xitun District, Taiwan), abbreviated here as L-FRP. The material density was 0.9 g/cc with a fiber density of 2.55 g/cc, initial fiber length of 25 mm, and diameter of 17 μm. The L-FRP pellets were dried for 4 h at 100 °C before use. The special ultra-long fiber injection machine (basic model CLF-180-TXL) used in this study was developed jointly with CLF (Chuan Lih Fa Machinery Works Co. Ltd., Tainan City, Guanmiao District, Taiwan). Details of the injection molding process and basic parameter setting used are shown in Table 1. The temperature profile along the barrel was 230 °C (melt temperature), the injection pressure was 70 bar, the injection time was 2 s, the injection speed was 60 mm/s, the cooling time was 15 s, the mold temperature was 70 °C, the back pressure was 3%, and the screw speeds used were 30 to 120 rpm. The screw speed had a significant effect on the final fiber length [20]. The molding machine had a total mold clamp force of 180 tons, a maximum injection speed of 150 mm/s, and a maximum injection pressure of 177 MPa. To retain as much of the original fiber length as possible, a three-stage injection unit was used with (i) a low breaking fiber plasticization unit (Ø 55 mm), (ii) an injection plunger (Ø 45 mm), and (iii) a packing plunger (Ø 40 mm), as shown in Figure 2. Each step involved a single operation and they did not interfere with each other, so that operations such as changing the screw of the conventional injection unit, as well as the forward and reverse functions, could be easily carried out. A reduction in the fiber breaking was expected in the injection molding stage, owing to less shearing during the plasticizing and molding procedures. The compression ratio of the plasticity screw in this study was lower than that of a conventional screw, and the fiber length that could be used was 1 to 30 mm. An accurate injection controller and procedure monitoring system were installed in order to ensure a stable molding process, plasticizing quality, and screw position control.

### 2.3. Experimental Design

**Figure 3:**
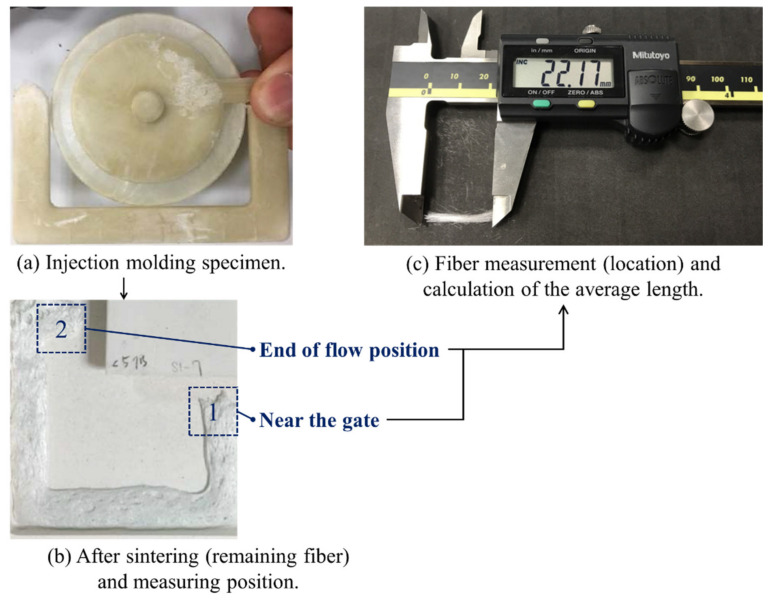
Schematics of the measurement position of the fiber length after breaking and the calculation of the fiber proportion: (**a**) injection molding part, (**b**) specimens were high-temperature sintering and leaving only the fibers that could then be measured, and (**c**) measurement fiber length.

Experiments were conducted to investigate the effects of the different mold cavity geometry, melt fill path, and plasticization parameters on the fiber orientation and melt fluidity for ultra-long glass fiber composite specimens, and to observe the change of fiber length by setting different screw speeds (from 30 to 120 rpm), which have a significant effect on the fiber length [20]. In addition, flow characteristic tests were carried out on 20 specimens under identical conditions, and the average value of the last 15 was used for analysis. The melt flow length and specimen weight were measured. The specimens were then volatilized by high-temperature sintering at 600 °C for 4 h, leaving only the fibers that could then be measured. The average length of the broken fibers was determined by the measurements of 200 individual fibers (see Figure 3). It was found that inappropriate settings of the parameters could result in the formation of defects such as fiber bundles and cold wells, which further degraded the mechanical properties of the specimens. The effects of the injection parameters on the properties of the L-FRP specimens have been investigated in many studies [14,15,16,17]. To better understand the effects of the melt fluidity and flow behavior of the mold cavity volume on the melt, many flow length specimens were tested, and Equation (1) was used to calculate the melt flow length ratio variations. In these experiments, the back pressure was set to 3%, the screw speed to 60 rpm, and the melt temperature to 230 °C. These parameters were defined as the standard molding criteria. The individual parameters of the melt flow path, gate design, and specimen thickness were then changed to investigate their effects on the flow behavior. In addition to this, the effects of different fiber lengths on the melt flow length were investigated by varying the screw speed. The changes in the fiber breaking length and melt flow length were studied at screw speeds of 30, 60, and 120 rpm. Changes in the back pressure and melt/mold temperature with respect to the melt flow length were also investigated. The correlated plasticization parameters of the flow behavior during the ultra-long fiber injection molding process are listed in Table 1.

## 3. Results and Discussion

### 3.1. Melt and Fiber Flow Behavior of the Fiber Length Variations

**Figure 4:**
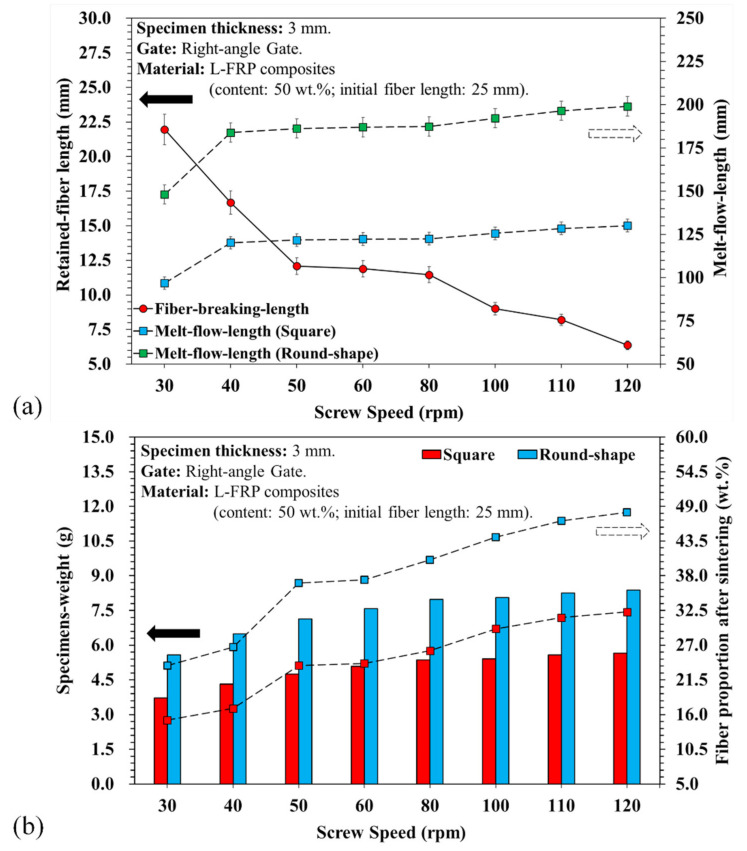
(**a**) Fiber breaking length variations of the melt flow length with different screw speeds and melt fill paths; (**b**) measurement of the average specimen original weight and calculation of the fiber-proportion after sintering.

Figure 4a presents a comparison of the fiber breaking lengths measured under different screw speeds. As the screw speed increased, so did the shear rate, which caused more fibers to break during the plasticization process and reduced the fiber length. However, the fiber length could be retained at 50% or longer (>15 mm average) during the plasticization process with a screw speed of 30~40 rpm. When the screw speed was increased to 50~80 rpm, the fiber length was reduced to around 45% (fiber length of 10 mm or longer). At a higher screw speed, the shear rate increased so much that there was a notable fracture with the fibers being less than 10 mm long, about 20% of their original length. On the other hand, the melt flow length increased with an increase in screw speed.

**Figure 5:**
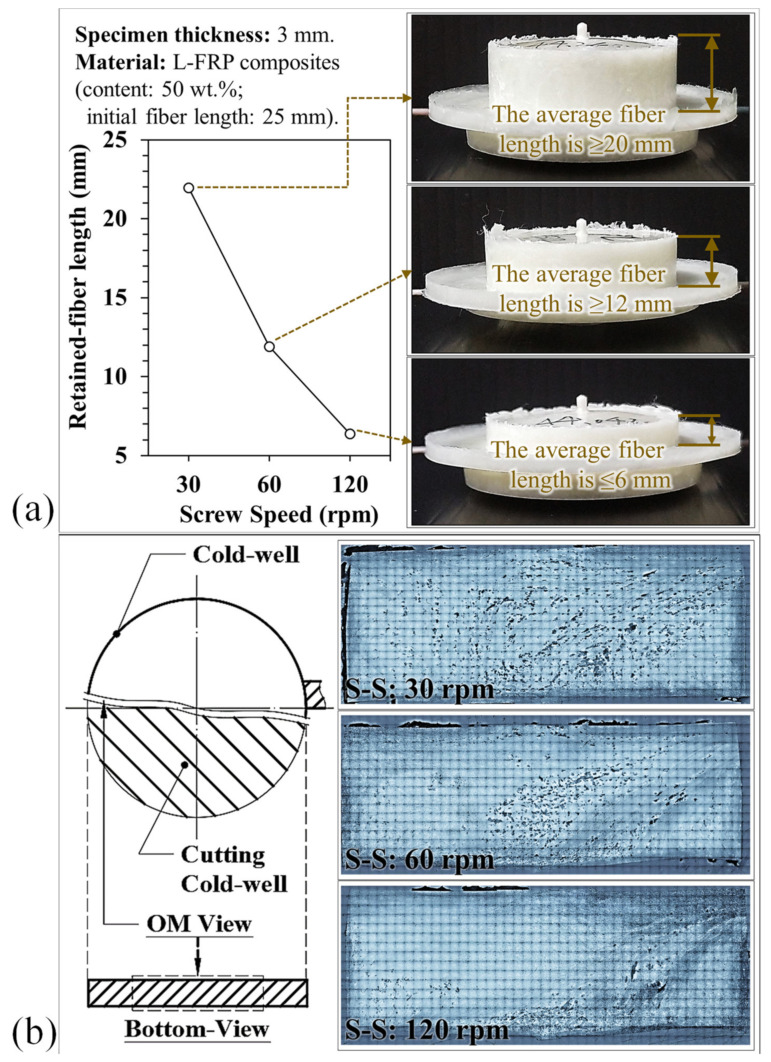
Optical scanning and observation of the cold well at different fiber breaking lengths (screw speeds of 30, 60, and 120 rpm) with the right-angle gate: (**a**) fiber stagnation, and (**b**) fiber flow and orientation.

A comparison of the square melt fill path with the spiral path showed serious differences. In the square melt fill path, fibers could easily become entangled, its mechanical properties were poor, and proper dispersion was difficult if not impossible. However, in the spiral melt fill path, there was less resistance to flow during the melt filling process, and there was an improvement in the melt flow ability as well as a longer melt flow length. Past research [20] has also shown that different kinds of flow paths affect the flow and dispersion of fibers into the mold cavity. The reason for this is that the flow of melt containing ultra-long fibers into the mold cavity is radial, and if the path is smooth the fibers will be easily dispersed. This phenomenon is also observed in the formation of shorter fibers, where faster screw speeds and fluent melt fill paths cause more fibers to break. The fibers exert a relatively low resistance for melting flow, and the result is an increase in flow length. Furthermore, the variations in the fiber breaking and length affected the flow ability between the fibers and melt, which resulted in differences in the weight and fiber proportion in the samples (see Figure 4b). The results show that the fiber lengths decreased with an increase in screw speed due to breaking, which enhanced the fluidity of the L-FRP composites. Many pores appeared in the cold well region at a screw speed of 30 rpm (see Figure 5a). This air trap porosity caused by longer fibers in the melt could not be successfully eliminated during the injection and molding stages (Figure 5b).

**Figure 6:**
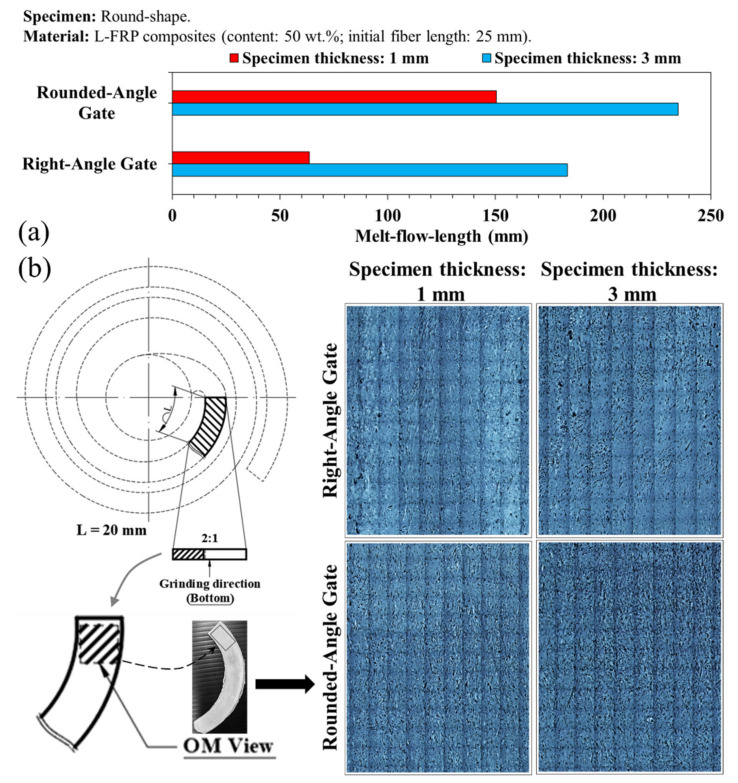
(**a**) Variations of melt flow length with different gate design and specimen thickness. (**b**) Optical scanning and observation of the fiber flow and orientation on the specimen.

**Figure 7:**
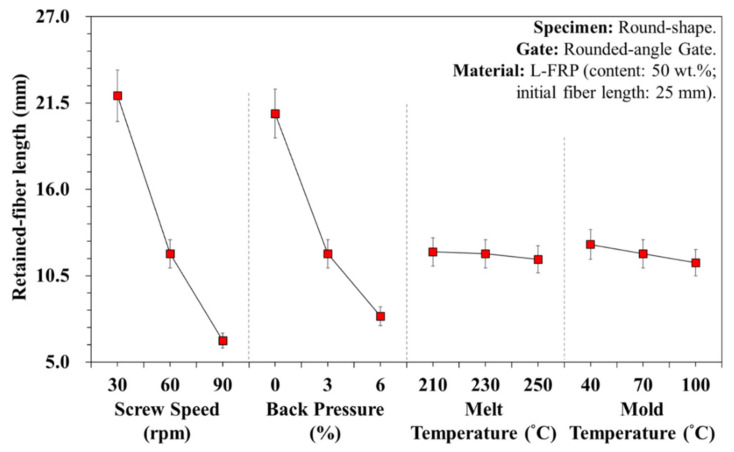
Variations of fiber breaking length under different plasticization parameters.

**Figure 8:**
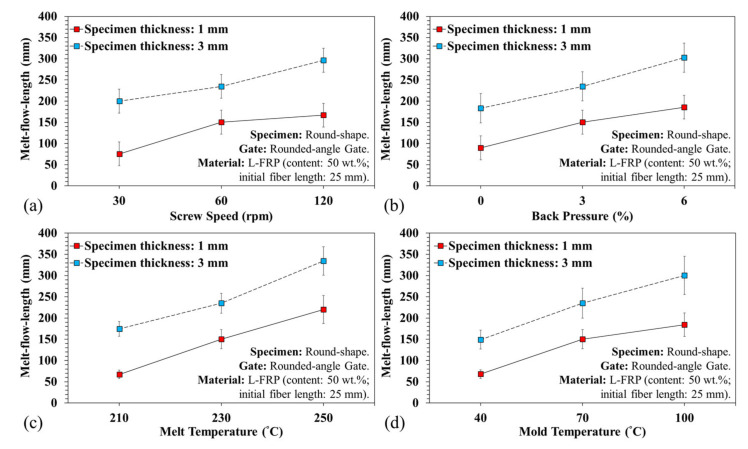
Variations of melt flow length under different specimen thicknesses and plasticization parameters: (**a**) variations of screw speed, (**b**) variations of back pressure, (**c**) variations of melt temperature, and (**d**) variations of mold temperature.

**Figure 9:**
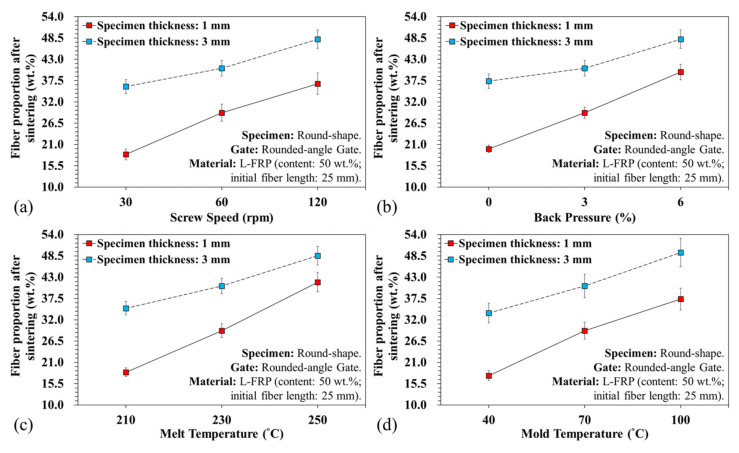
Variations of the fiber proportions under different specimen thicknesses and plasticization parameters: (**a**) variations of screw speed, (**b**) variations of back pressure, (**c**) variations of melt temperature, and (**d**) variations of mold temperature.

Figure 6 shows the variations of the melt flow ability with different gate designs and specimen thicknesses. It is clear that gate design has an effect on the melt flow length. The flow ability was greatly improved by the large filling channel, which also increased the flow length. The experimental results revealed that the mold cavity volume and thickness had a substantial influence on the melt flow ability of the fiber containing the melt during the filling process. In addition, when the specimen was thin, an increase in the thickness of the frozen layer (the skin) hindered melt filling, resulting in a short melt flow length. In contrast, the amount of air trap porosity may have decreased with an increase in screw speed, and the fluent melt fill path also enhanced mixability, despite the reduced fiber length (see Figure 6b). The fiber breaking and flow ability for both the rounded and square shaped gates were investigated at different screw speeds of 30, 60, and 120 rpm. These experiments gave insight into the effects of the gate design and specimen thicknesses on the melt flow ability. The effects of back pressure and melt/mold temperature on the fiber breaking variation, melt flow ability, and fiber proportion were also investigated (see Figure 7, Figure 8 and Figure 9).

### 3.2. Plasticization Parameter Variations and Melt and Fiber Flow Behavior

Figure 7 shows the breaking variations of the fiber length with different plasticization parameters. It is clear that the screw speed and back pressure affect the fiber length. Shear rate was greater at high screw speeds and with back pressure, and the result was shorter fibers. The experimental results revealed that the pressure and shear rate in the plasticizing process had a substantial influence on fiber breaking during the plasticization and molding stage—the shorter the relative fiber length, the lower the flow resistance to the melt, and the longer the relative melt flow length (Figure 8a,b). Increasing the melt and mold temperature improved the fluidity (Figure 8c,d), but did not result in shorter fibers. This is because during the fiber filling process, increasing the temperature facilitated the flow of fibers, resulting in less breakage. This increased both the fiber diffusivity and melt flow length. However, the screw speed and the back pressure were maintained at the same plasticization parameters (screw speed of 60 rpm and back pressure of 3%), so the fiber length obtained was between 11 and 13 mm on average. Figure 8 and Figure 9 illustrate the variations of the melt flow length and fiber proportion at different specimen thickness and plasticization parameters. At a high screw speed, the shear increased and more fibers broke. However, during filling, a composite with short fibers had less resistance to flow and, consequently, the melt flow length and fiber proportion increased.

An increase in back pressure also resulted in increased mixability of the melt and fibers, but the amount of fibers breaking rose. Consequently, the shorter fibers lowered the flow resistance of the melt, which increased the proportion of fibers flowing into the mold cavity. In addition, the fiber and melt flow ability in relation to the melt and mold temperature resulted in an increased flow ability. During the filling process, the melt temperature fiber containing plastic materials could be affected by the cooling channel of the mold, and the melt could be frozen, further affecting the flow resistance. When the low temperature melt approached the mold cavity surface, it could be frozen by the cooling channel. This cooling phenomenon is more prevalent in thin products.

## 4. Conclusions

An ultra-long fiber composite injection molding machine (with a three-stage plunger and low breaking fiber mechanism) was used to conduct injection molding experiments under different mold cavity geometry and plasticization parameters. The notable research results are as follows:

- A low breaking fiber mechanism in a specially designed injection molding system was invented that could retain the fiber length to a significant degree. Specifically, a fiber length of about 22 mm could be retained from an input of L-FRP with a fiber length of 25 mm. A relationship between tensile stress and the changes of the fiber length of the material (L-FRP) with the operational changes was elucidated.
- In this study, for the melt flow ability experiments with different gate designs and specimen thicknesses, a rounded-angle gate design and thicker specimen thickness decreased the flow resistance and provided a better melt flow length for the L-FRP injection molding.
- Screw speed and fiber breakage were directly related. At a low screw speed, 50% of the fiber length was retained. At a high screw speed, the shear rate rose, and broken fibers were 10 mm or less in length. This was 20% of the original length. An increase in the screw speed and back pressure resulted in a decrease in fiber length and flow resistance. The cooling channels froze the melt with long fibers and at low temperatures as the melt approached the mold cavity surface. The freezing phenomenon was more prevalent for parts with thin walls, which in turn reduced the melt fluidity and fiber proportion in the specimens.
- The high mechanical performance characteristics of the long fiber composites are the reason they are often selected as substitutes for metals, as a replacement for under-performing plastics, or as alternatives to higher-cost engineering polymers through the up-engineering of lower-cost plastics. Based on the results of this research, the length of the fibers could be controlled by the setting of the screw speed, which could be applied in the manufacturing of automobile and airplane components that need to have different fiber lengths and geometric products, or those that need a high tensile strength and tensile modulus.

## Figures and Tables

**Table 1:** Plasticization parameters for the manufacture of the injection molded L-FRP spiral flow test specimens.

Basic Parameter Setting							
Injection Pressure (bar)		70		Screw Speed (rpm)		60	
Injection Speed (mm/s)		60		Back Pressure (%)		3	
Injection Time (s)		2		Melt Temperature (°C)		230	
Cooling Time (s)		15		Mold Temperature(°C)		70	
Random Order of Treatment of the Molding Samples							
Method	Screw speed (rpm)		Back pressure (%)		Melt temperature (°C)		Mold temperature (°C)
1	30		3		230		70
2	60		3		230		70
3	120		3		230		70
4	60		0		230		70
5	60		6		230		70
6	60		3		210		70
7	60		3		250		70
8	60		3		230		40
9	60		3		230		100

## Data Availability

The data presented in this study are available on request from the corresponding author.
